# Human umbilical cord mesenchymal stem cell-derived exosomal miR-335-5p attenuates the inflammation and tubular epithelial–myofibroblast transdifferentiation of renal tubular epithelial cells by reducing ADAM19 protein levels

**DOI:** 10.1186/s13287-022-03071-z

**Published:** 2022-07-28

**Authors:** Zhenhua Qiu, Zhihui Zhong, Yuehan Zhang, Haoling Tan, Bo Deng, Guohuang Meng

**Affiliations:** Department of Laboratory Medicine, The People’s Hospital of Gaozhou, Maoming, 525200 China

**Keywords:** Chronic kidney disease, Exosome, miRNA, Renal fibrosis

## Abstract

**Background:**

Renal tubular epithelial–myofibroblast transdifferentiation (EMT) plays a key role in the regulation of renal fibrosis. Exosomes derived from human umbilical cord mesenchymal stem cells (hucMSCs) play a crucial role in alleviating renal fibrosis and injury. Additionally, hucMSC-derived exosomes contain numerous microRNAs (miRNAs). However, it is unclear whether mesenchymal stem cells can regulate the transforming growth factor (TGF)-β1-induced EMT of human renal tubular epithelial cells (RTECs) through exosomal miRNAs.

**Method:**

HK-2, a human RTEC line, was co-treated with TGF-β1 and hucMSC-derived exosomes. Additionally, TGF-β1-treated HK-2 cells were transfected with a miR-335-5p mimic and disintegrin and metalloproteinase domain-containing protein 19 (ADAM19)-overexpression plasmid. miR-335-5p expression and ADAM19 protein and inflammation levels were measured via quantitative reverse transcription polymerase chain reaction, western blotting, and enzyme-linked immunosorbent assays, respectively.

**Results:**

TGF-β1 treatment changed the shape of HK-2 cells from a cobblestone morphology to a long spindle shape, accompanied by an increase in interleukin (IL)-6, tumor necrosis factor-α, IL-1β, collagen I, collagen III, α-smooth muscle actin, vimentin, and N-cadherin protein levels, whereas E-cadherin protein levels were reduced in these HK-2 cells, suggesting that TGF-β1 treatment induced the inflammation and EMT of HK-2 cells. HucMSC-exosomes improved the inflammation and EMT phenotype of TGF-β1-induced HK-2 cells by transferring miR-335-5p. miR-335-5p was found to bind the *ADAM19* 3′-untranslated region to reduce ADAM19 protein levels. Additionally, miR-335-5p improved the inflammation and EMT phenotype of HK-2 cells by reducing ADAM19 protein levels with TGF-β1 induction.

**Conclusions:**

HucMSC-derived exosomal miR-335-5p attenuates the inflammation and EMT of HK-2 cells by reducing ADAM19 protein levels upon TGF-β1 induction. This study provides a potential therapeutic strategy and identifies targets for clinically treating renal fibrosis.

## Introduction

Chronic kidney disease, primarily induced by chronic pyelonephritis and glomerular diseases, diabetes, or hypertension, affects approximately 10% of the global population [1, 2]. The kidney function of patients with chronic kidney disease is lost progressively over time, often resulting in end-stage renal disease and advancing to kidney failure and even death [2]. Renal fibrosis is the common pathway underlying most progressive nephropathy, including excessive accumulation of the extracellular matrix, and its histological manifestations include loss of the capillary network, the aggregation of fibrous collagen, activated myofibroblasts, and inflammatory cells [3–5]. Transforming growth factor-β1 (TGF-β1) is considered a key pro-fibrotic drug for renal fibrosis [6]. The TGF-β1/Smad signaling pathway causes inflammation, renal tubular epithelial–myofibroblast transdifferentiation (EMT), and abnormal extracellular matrix deposition, which is a key pathway in the induction of renal fibrosis [7]. The EMT process is often accompanied by the overexpression of mesenchymal cell markers such as α-smooth muscle actin (α-SMA), vimentin, and N-cadherin and the downregulation of epithelial cell markers, such as E-cadherin [8]. A previous study found α-SMA, vimentin, and N-cadherin overexpression and E-cadherin downregulation in TGF-β1-treated human RTECs, accompanied by enhanced collagen I and collagen III protein levels [9]. Collagen I and collagen III protein overexpression leads to cellular matrix deposition during the process of renal fibrosis. However, the inhibition of EMT can contribute to improved renal fibrosis [10, 11]. Although substantial progress has been made in understanding renal fibrogenesis, there is no effective therapy to reverse renal fibrosis progression or restore renal tissue injury. Therefore, new treatment strategies need to be developed.

The prospects for regenerative medicine have aroused widespread interest in stem cell therapy. Stem cells have become a promising strategy for reversing renal fibrosis progression [12, 13]. Human umbilical cord mesenchymal stem cells (hucMSCs) have better proliferation ability and lower immunogenicity than bone marrow MSCs, making these ideal therapeutic stem cells. Studies have shown that intravenously injected hucMSCs can inhibit activation of the TGF-β1/Smad axis to reduce renal fibrosis and extracellular matrix deposition [14, 15]. However, the in vivo injection of stem cells requires large number of cells. When stem cells proliferate in vitro, uncontrolled growth, poor cell expansion, and senescence occur. Moreover, the immunogenicity of stem cells can cause rejection by the recipient’s immune system, leading to limitations in stem cell therapy. Exosomes are small membrane vesicles (40–100 nm in diameter) that can transfer protein and microRNA (miRNA) to target cells to play a regulatory role and mediate crosstalk between cells. HucMSC-exosomes can improve cardiac and epidural fibrosis [16–18]. At the same time, hucMSC-exosomes can reduce cisplatin-mediated renal injury, ameliorating renal interstitial fibrosis in the later period [19, 20]. By silencing the TLR4/NF-κB signaling pathway, the secretion of inflammatory factors and the deposition of extracellular matrix were reduced, and renal fibrosis in the unilateral ureteral obstruction rat model was improved after the intravenous injection of the serum-free medium of HucMSCs. [21]. However, to date, the effect of hucMSC-exosomes on renal fibrosis and the underlying mechanisms are still not fully understood.

miRNA regulates the expression of target genes by inhibiting the translation of transcription products, thereby regulating important biological processes, including renal fibrosis. The upregulation miR-29 and miR-200 or downregulation of miR-21 and miR-192 can reduce TGF-β1/Smad3-mediated renal fibrosis [6]. miR-9-5p prevents TGF-β1 from inducing fibrosis in human renal proximal tubular epithelial cells [22]. miR-135a-5p promotes EMT in HK-2 cells, a human renal proximal tubular epithelial cell line, induced by the TGF-β1-mediated targeting of SIRT1 [23]. However, it is unclear whether mesenchymal stem cells can regulate TGF-β1 expression to induce the EMT of HK-2 cells through exosomal miRNAs.

In this study, TGF-β1 was used to treat HK-2 cells. Next, the cell phenotype, protein levels of collagen I, collagen III, E-cadherin, α-SMA, vimentin, and N-cadherin, and the levels of tumor necrosis factor-α (TNF-α), interleukin 6 (IL-6), and IL-1β were measured to evaluate the regulatory effect of TGF-β1 on EMT and inflammatory phenotypes in HK-2 cells. Next, we aimed to evaluate the regulatory effect of hucMSC-exosomal miRNAs on EMT and inflammation using TGF-β1-induced HK-2 cells as well as to determine the associated mechanism.

## Materials and methods

### Cell culture and treatment

HK-2 cells, human RTEC line, were cultured in DMEM/F12 medium containing 10% fetal bovine serum (FBS) at 37 °C with 5% CO_2_ and then cultured under serum-starved conditions overnight and incubated with 10 ng/ml recombinant human TGF-β1 (R&D Systems, Minneapolis, MN, USA) at 24 h to induce the EMT of HK-2 cells [22]. hucMSCs were cultured in α-MEM containing 10% FBS at 37 °C with 5% CO_2_.

### Exosome collection and identification

For exosome collection, hucMSCs were cultured for 48 h in exosome-free α-MEM medium without serum, and the conditioned media were collected to isolate exosomes using total exosome isolation reagent (GENESEED, Guangzhou, China). The morphology, particle number and size, and exosomal biomarker CD81 protein and cell biomarker β-actin protein were examined using a Hitachi H-7650 transmission electron microscope (TEM, Tokyo, Japan), the ZetaView® Nanoparticle Tracking Analysis (NTA) instrument (Particle Metrix, Meerbusch, Germany), and western blotting, respectively. Finally, HK-2 cells were treated with TGF-β1 for 24 h and then TGF-β1-induced HK-2 cells were treated one-time 10, 20, and 40 µg protein-equivalent of hucMSC-derived exosomes for 24 h to analyze the effect of exosomes on the EMT and inflammation in TGF-β1-induced HK-2 cells.

### Reverse transcription polymerase chain reaction (RT-qPCR)

Total RNA was isolated using 1 ml of TRIzol reagent (Invitrogen). Next, a reverse transcription reaction to obtain cDNA was carried out according to the method of the PrimeScript™ RT reagent Kit (Takara, Dalian). For qPCR, miR-335-5p expression in HK-2 cells after TGF-β1 treatment was detected using the Mir-X miRNA qPCR SYBR Kit (Clontech Laboratories, Inc., USA) in a 7500 Real-Time PCR System (Applied Biosystems, Foster City, CA, USA). U6 served as the reference gene. The relative expression of miRNAs was calculated using the 2^−ΔΔCT^ method. The primer sequences were as follows: miR-335-5p forward, 5′-ACACTCCAGCTGGGCAAAGTGCTTACAGTGC-3′ and reverse, 5′-CTCAACTGGTGTCGTGGA-3′; U6 forward, 5′-CTCGCTTCGGCAGCACA-3′ and reverse, 5′-AACGCTTCACGAATTTGCGT-3′. All experiments were performed with three independent replicates.

### Western blotting

Western blotting was performed as described previously [24]. Briefly, total proteins were collected, quantified, and separated using 10% SDS-PAGE. The proteins were then transferred onto a polyvinylidene fluoride membrane (Millipore, Billerica, MA, USA) and blocked with 5% milk. Next, the membrane was incubated with primary antibodies, followed by a horseradish peroxidase-conjugated secondary antibody, and visualized. The following primary antibodies were used: anti-ADAM19 antibody (ab191457), anti-collagen I antibody (ab34710), anti-collagen III antibody (ab184993), anti-α-SMA antibody (ab5831), anti-vimentin antibody (ab92547), anti-N-cadherin antibody (ab76011), anti-E-cadherin antibody (ab40772), anti-GAPDH antibody (ab8245). Secondary antibodies were as follows: goat anti-rabbit IgG(H + L) and mouse/human ads-HRP (ab6702). Antibodies were purchased from Abcam (Cambridge, MA, USA). Enhanced chemiluminescent reagent (Thermo Scientific Pierce, Rockford, IL, USA) was used to visualize the protein abundance, and the grayscale of protein bands was analyzed using ImageJ 6.0. All experiments were performed with three independent replicates.

### Enzyme-linked immunosorbent assay (ELISA)

The protein levels of IL-4, IL-10, TNF-α, IL-6, and IL-1β in the conditioned media of HK-2 cells were detected using ELISA Kits (bsk11004, bsk11010, bsk11014, bsk11007, bsk11001, Bioss, Beijing, China) according to the test kit instructions. All experiments were performed with three independent replicates.

### Cell transfection

The open reading frame of disintegrin and metalloproteinase domain-containing protein 19 (*ADAM19*) was synthesized by GENEWIZ (Suzhou, China) and linked into pcDNA 3.1 (ov-ADAM19), and the empty pcDNA 3.1 was used as a negative control (ov-NC). The miR-335-5p mimic and inhibitor, NC mimic/inhibitor, si-ADAM19, and si-NC were purchased from RiboBio (Guangzhou, China). Cell transfections were performed using Lipofectamine 3000 (Invitrogen).

### Dual-luciferase reporter assay

The wild-type (WT) and mutant type (MUT) 3ʹ untranslated regions (3ʹ UTRs) of *ADAM19* were synthesized (GENEWIZ, Suzhou, China) and linked into the luciferase reporter vector psi-CHECK-2 (Promega). HK-2s cells were co-transfected with the psi-CHECK-2-ADAM19 sequence and miR-335-5p mimic. After 48 h of transfection, firefly and *Renilla* luciferase activities were determined using a Dual-Luciferase Reporter Assay System (Promega). All experiments were performed based on three independent replicates.

### Statistical analysis

SPSS software (version 19.0; IBM, Chicago, IL, USA) was used to analyze the data normality and differences. All normality data are presented as the mean ± standard deviation. Differences between two groups were analyzed using a student's *t* test if data conformed to a normal distribution. Differences between three or more groups were analyzed using one-way analysis of variance (ANOVA), followed by Tukey’s multiple comparisons test. Statistical significance was set at *P* < 0.05.

## Results

### HucMSC-exosomes diminish EMT and inflammation in HK-2 cells induced by TGF-β1 treatment

**Fig. 1:**
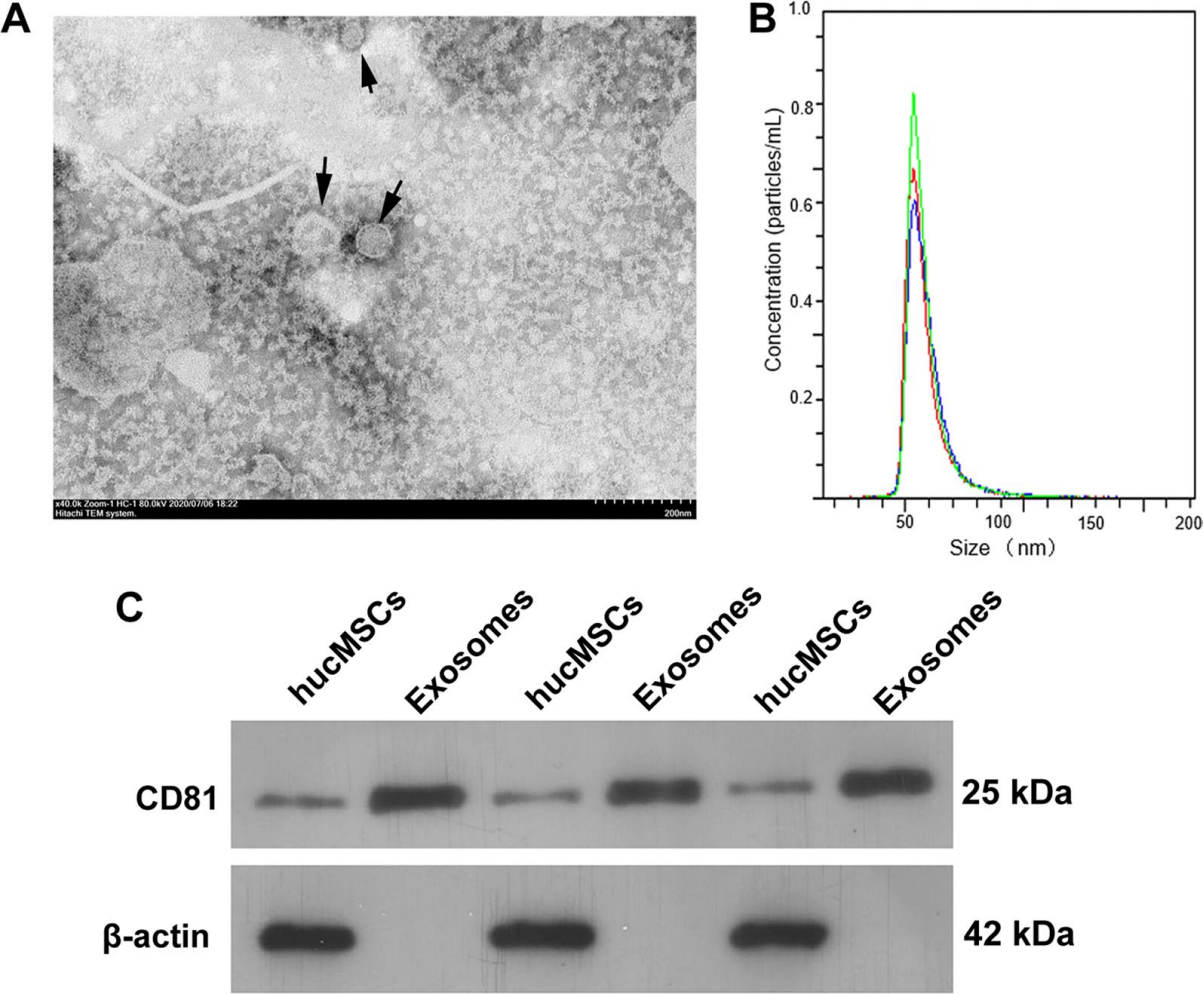
Identification of human umbilical cord mesenchymal stem cell (hucMSC)-exosomes. **A** hucMSC-exosomes were identified by transmission electron microscopy. **B** The size distribution of hucMSC-exosomes was measured by Nanoparticle Tracking Analysis. **C** CD81 and β-actin in hucMSC-exosomes were measured via western blotting (*n* = 3)

**Fig. 2:**
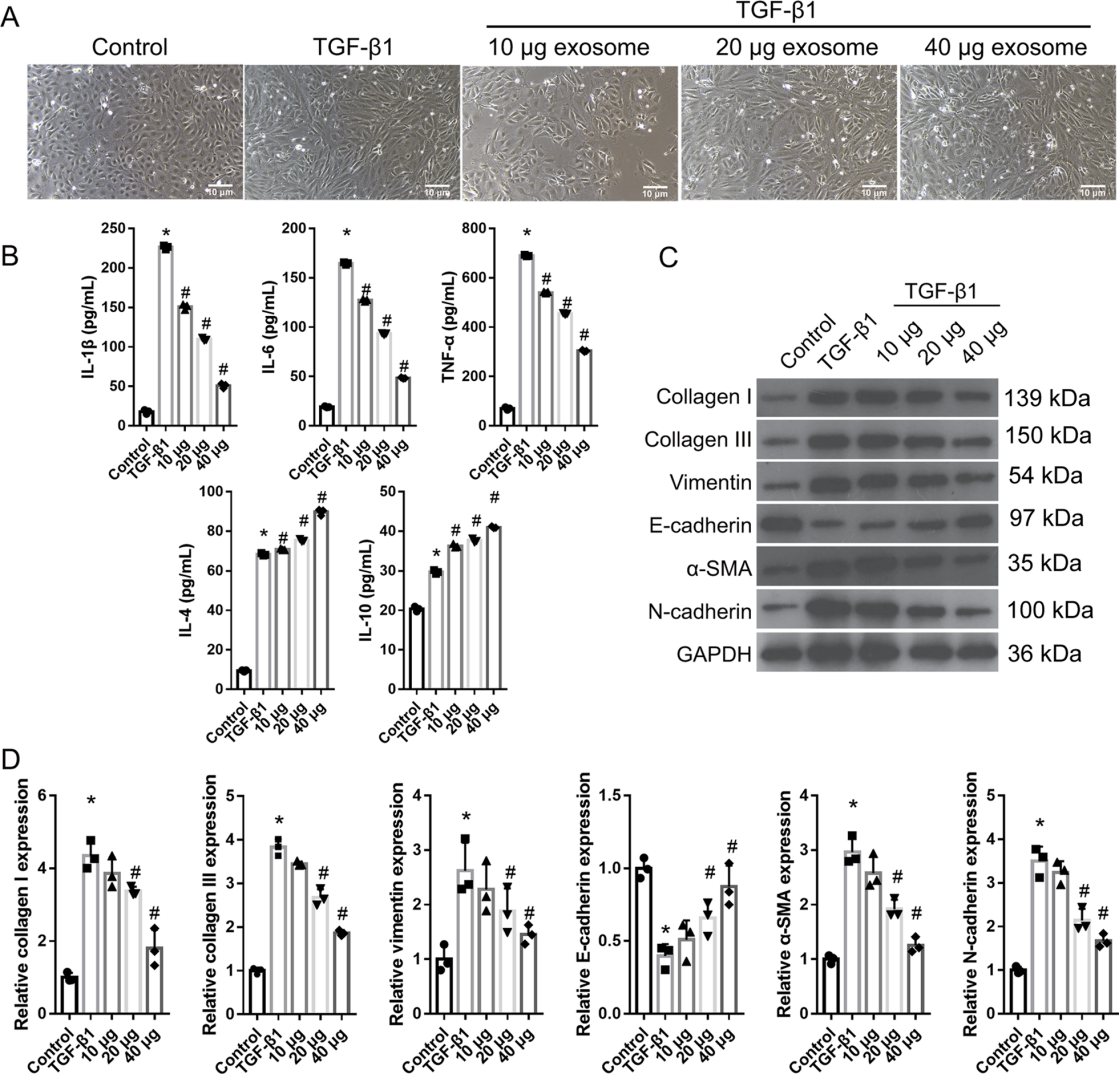
Human umbilical cord mesenchymal stem cell (hucMSC)-exosome treatment can reverse the effects of TGF-β1 on the epithelial–myofibroblast transdifferentiation (EMT) and inflammation of HK-2 cells. **A** The shape of TGF-β1-treated HK-2 cells returned to a normal HK-2 cell morphology after hucMSC-exosome treatment in TGF-β1-treated HK-2 cells. **B** The levels of TNF-α, IL-6, and IL-1β were decreased whereas the levels of IL-4 and IL-10 were increased after hucMSC-exosome treatment of TGF-β1-treated HK-2 cells (*n* = 3). **C** The protein levels of collagen I, collagen III, α-SMA, vimentin, and N-cadherin in TGF-β1-induced HK-2 cells were decreased, whereas those of E-cadherin were increased after hucMSC-exosome treatment in TGF-β1-treated HK-2 cells. **D** Statistical results of protein expression (*n* = 3). (*P < 0.05, vs control group; #*P* < 0.05, vs TGF-β1-treated group). In TGF-β1 + 10, 20, and 40 µg exosome groups, HK-2 cells were treated with TGF-β1 for 24 h and then TGF-β1-induced HK-2 cells were treated one-time 10, 20, and 40 µg protein-equivalent of hucMSC-derived exosomes for 24 h. All statistical analyses were performed by one-way ANOVA, followed by Tukey’s multiple comparisons post hoc test

First, exosomes were extracted, purified, and identified from the supernatant of hucMSC medium. The TEM and NTA results showed that the exosomes had a typical dish-shaped double-layer membrane structure, with a diameter of 50–150 nm. CD81, a marker of exosomes, was highly expressed in these exosomes. These results indicated that the exosomes were successfully purified (Fig. 1). To determine whether hucMSC-exosomes could prevent the EMT and inflammation in HK-2 cells, the exosomes were co-cultured with TGF-β1-induced HK-2 cells. Normal HK-2 cells had a cobblestone morphology (round or oval); TGF-β1-induced HK-2 cells showed a long spindle shape, and hucMSC-exosome treatment resulted in most TGF-β1-induced HK-2 cells having a normal HK-2 cell morphology (Fig. 2A). Compared with those in the normal HK-2 group, the levels of IL-4, IL-10, TNF-α, IL-6, and IL-1β and the protein levels of collagen I, collagen III, α-SMA, vimentin, and N-cadherin in the TGF-β1-induced HK-2 group were significantly increased, whereas E-cadherin levels were significantly decreased (Fig. 2B–D). Additionally, compared with those in the TGF-β1-induced HK-2 group, the levels of TNF-α, IL-6, and IL-1β and the protein levels of collagen I, collagen III, α-SMA, vimentin, and N-cadherin in the hucMSC-exosome treatment group were significantly decreased, whereas IL-4, IL-10, and E-cadherin levels were significantly increased (Fig. 2B–D). hucMSC-exosome treatment reversed the effect of TGF-β1 on HK-2 cells in a concentration-dependent manner (Fig. 2B–D).

### miR-335-5p expression in hucMSC-exosomes and TGF-β1-induced HK-2 cells

**Fig. 3:**
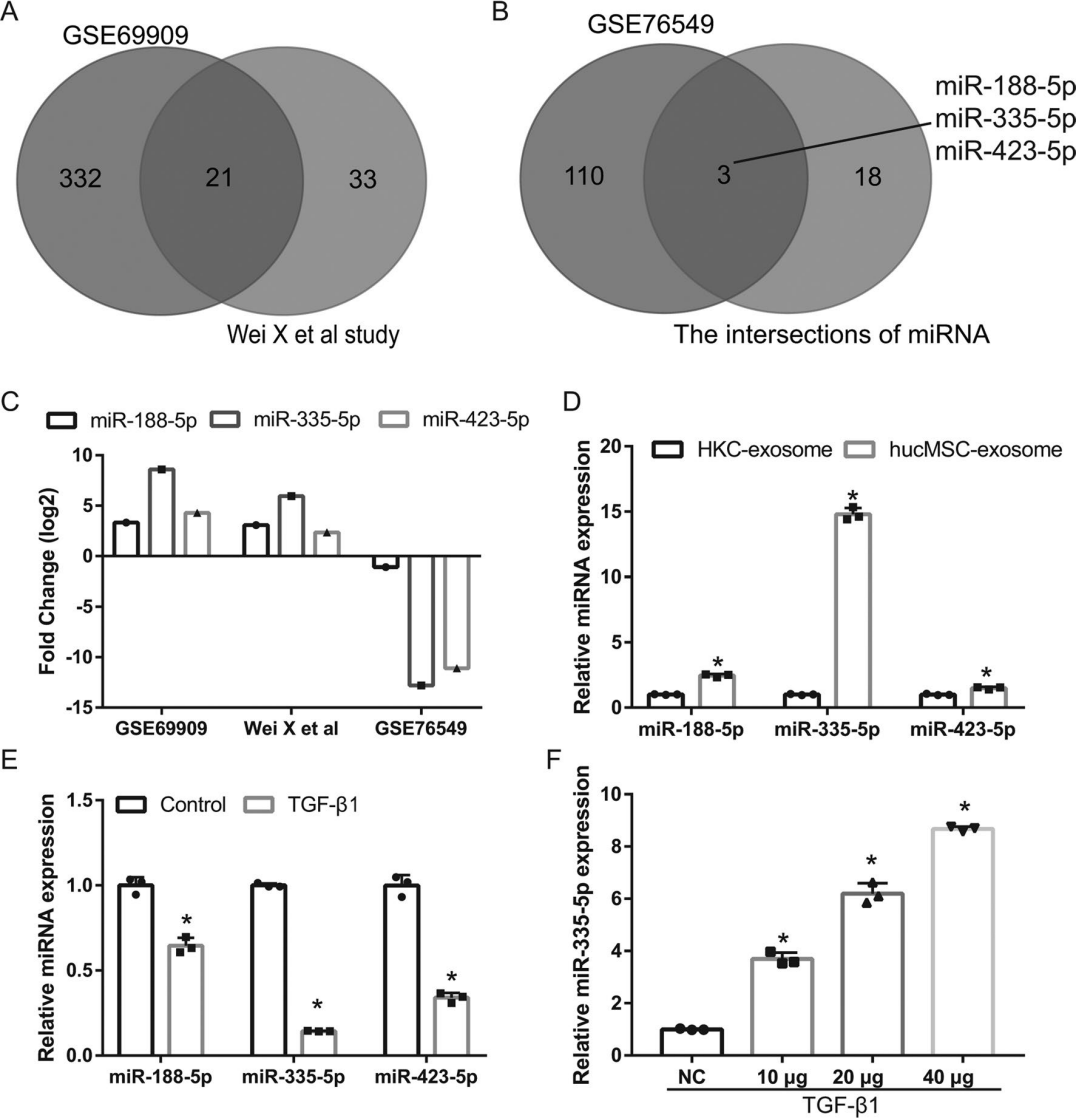
miR-335-5p expression is promoted by human umbilical cord mesenchymal stem cell (hucMSC)-exosome treatment in TGF-β1-treated HK-2 cells. **A** Venn image showing the overlapping miRNAs in hucMSC-exosomes between GSE69909 and Wei X results. **B** Venn image showing the overlapping miRNAs between miRNAs in hucMSC-exosomes and in kidney fibrotic tissues. **C** The expression difference (fold-change) of miR-188-5p, miR-335-5p, and miR-423-5p is shown according to GSE69909, Wei X results, and GSE76549. **D** The expression of miR-188-5p, miR-335-5p, and miR-423-5p was measured by RT-qPCR in hucMSC-exosomes. Statistical analyses were performed by student's *t* test (*n* = 3). **E** The expression of miR-188-5p, miR-335-5p, and miR-423-5p was measured by RT-qPCR in TGF-β1-treated HK-2 cells. Statistical analyses were performed by student's *t* test (*n* = 3). **F** miR-335-5p expression was measured by RT-qPCR after hucMSC-exosome treatment in TGF-β1-treated HK-2 cells (*n* = 3). Statistical analyses were performed by one-way ANOVA, followed by Tukey’s multiple comparisons post hoc test. (*P < 0.05)

To illustrate the mechanism by which hucMSC-exosomes regulate the EMT of HK-2 cells, miRNAs enriched in hucMSC-exosomes and significantly downregulated miRNAs in renal fibrotic tissue were analyzed. The GSE69909 dataset showed that compared to that in 293 T cell exosomes, 353 miRNAs were significantly enriched in hucMSC-exosomes. Compared with that in exosomes derived from human fetal lung fibroblast cells, 54 miRNAs were significantly enriched in hucMSC-exosomes [25]. By comparing miRNAs in exosomes of 293 T cell and human fetal lung fibroblast cells, unique miRNAs in hucMSC-exosomes could be found. Two sets of upregulated miRNAs intersected, and 21 upregulated miRNAs were identified in hucMSC-exosomes (Fig. 3A). The GSE76549 dataset showed that compared with that in normal mouse kidneys, the expressions of 113 miRNAs were significantly downregulated in kidney fibrotic tissues, which were intersected with 21 upregulated miRNAs in hucMSC-exosomes to obtain miR-188-5p, miR-335-5p, and miR-423-5p (Fig. 3B). The fold differences in the expression of miR-188-5p, miR-335-5p, and miR-423-5p are shown in Fig. 3C, which indicated that miR-335-5p exhibited the largest fold-change. Compared with those in the normal cultured HK-2-exosomes, the expression levels of miR-188-5p, miR-335-5p, and miR-423-5p, especially miR-335-5p, were significantly increased in hucMSC-exosomes (Fig. 3D). Compared with those in normal cultured HK-2 cells, the expression levels of miR-188-5p, miR-335-5p, and miR-423-5p in TGF-β1-induced HK-2 cells were significantly reduced, of which miR-335-5p was the most downregulated (Fig. 3E). Therefore, we selected miR-335-5p for follow-up research. Compared with that in the NC group, the expression of miR-335-5p in co-cultures with hucMSC-exosomes was significantly increased in HK-2 cells in a concentration-dependent manner (Fig. 3F).

### Reduction of hucMSC-exosomal miR-335-3p transport reduces the ameliorating effect of hucMSC-exosomes on EMT and inflammation in HK-2 cells

**Fig. 4:**
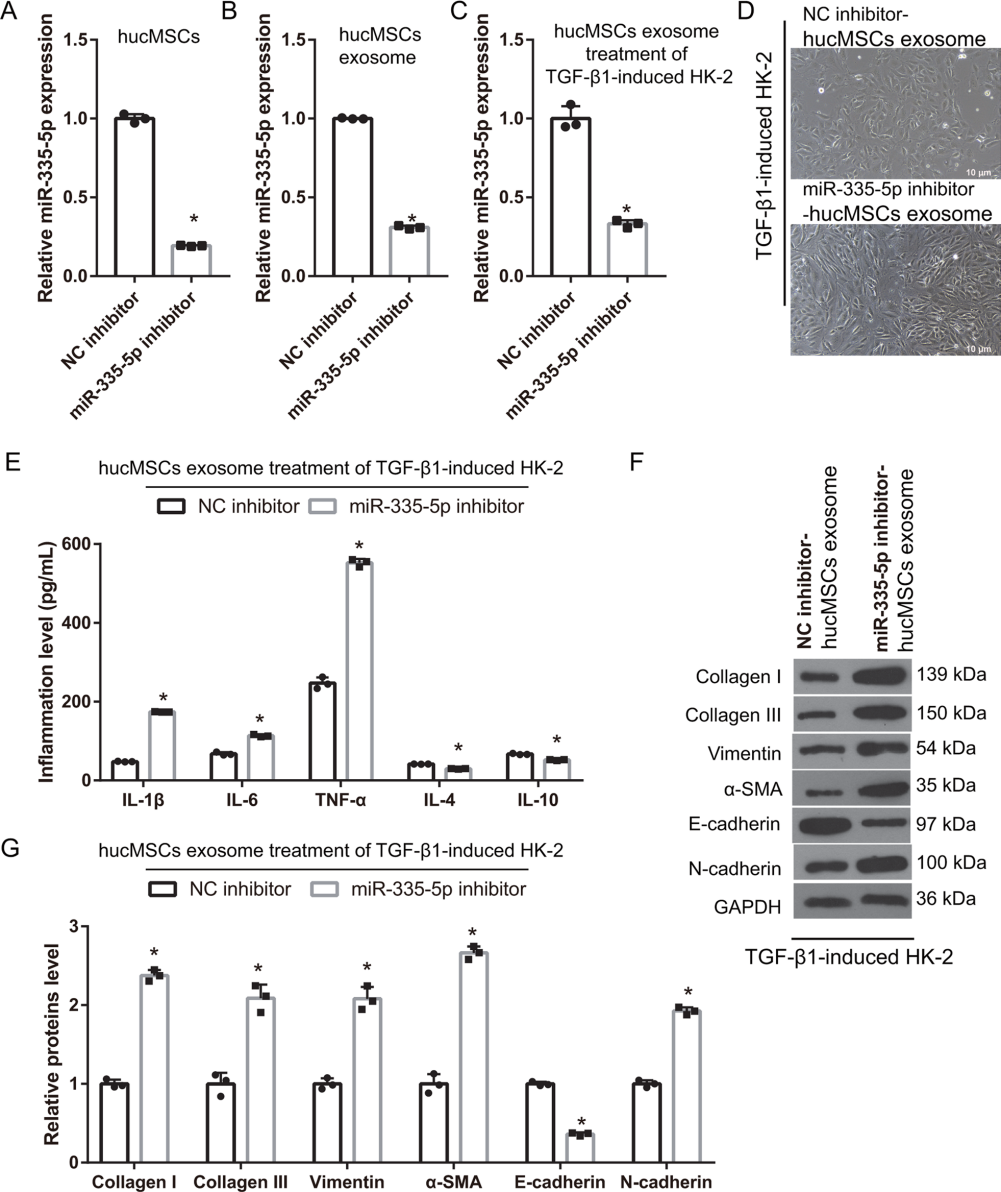
miR-335-5p silencing in human umbilical cord mesenchymal stem cells (hucMSCs) can reduce the effect of hucMSC-exosomes on the epithelial–myofibroblast transdifferentiation (EMT) and inflammation of TGF-β1-induced HK-2 cells. **A**, **B** miR-335-5p expression was measured by RT-qPCR in hucMSCs (**A**) and in hucMSC-exosomes (**B**) after transfection (*n* = 3). **C** miR-335-5p expression was measured by RT-qPCR in TGF-β1-treated HK-2 cells after treatment with 40 μg hucMSC-exosomes from NC inhibitor and miR-335-5p inhibitor groups (*n* = 3). **D** The shape of TGF-β1-treated HK-2 cells was detected after treatment with hucMSC-exosomes from NC inhibitor and miR-335-5p inhibitor groups. **E** The protein levels of TNF-α, IL-6, and IL-1β were increased whereas the levels of IL-4 and IL-10 were decreased in the miR-335-5p inhibitor group of TGF-β1-treated HK-2 cells treated with hucMSC-exosomes from the NC inhibitor and miR-335-5p inhibitor groups (*n* = 3). **F** The protein levels of collagen I, collagen III, α-SMA, vimentin, E-cadherin, and N-cadherin in TGF-β1-induced HK-2 cells were measured by western blotting after treatment with hucMSC-exosomes from NC inhibitor and miR-335-5p inhibitor groups (*n* = 3). **G** The statistical results of protein expression (*P < 0.05). In all groups, HK-2 cells were treated with TGF-β1 for 24 h and then TGF-β1-induced HK-2 cells were treated one-time 40 µg protein-equivalent of transfected-hucMSC-derived exosomes for 24 h. All statistical analyses were performed by student's *t* test

To understand the function of exosomal miR-335-5p in the EMT of HK-2 cells induced by TGF-β1, the miR-335-5p inhibitor was transfected into hucMSCs. miR-335-5p expression in hucMSCs and hucMSC-exosomes in the miR-335-5p inhibitor group was significantly lower than that in the NC inhibitor group (Fig. 4A, B). Next, the TGF-β1-treated HK-2 cells were treated with 40 μg of hucMSC-exosomes. Compared with that in the hucMSC-exosome treatment plus NC inhibitor group, miR-335-5p expression was significantly reduced in hucMSC-exosome-treated TGF-β1-induced HK-2 cells of the miR-335-5p inhibitor group (Fig. 4C). The shape of TGF-β1-induced HK-2 cells with hucMSC-exosome treatment in the NC inhibitor group was cobblestone, whereas that of TGF-β1-induced HK-2 cells with hucMSC-exosome and miR-335-5p inhibitor treatment was long spindles (Fig. 4D). The protein levels of TNF-α, IL-6, IL-1β, collagen I, collagen III, α-SMA, vimentin, and N-cadherin in TGF-β1-treated HK-2 cells were significantly increased, whereas IL-4, IL-10, and E-cadherin protein was significantly decreased, in the miR-335-5p inhibitor group (Fig. 4E–G).

### miR-335-5p diminishes EMT and inflammation in HK-2 cells induced by TGF-β1 treatment

**Fig. 5:**
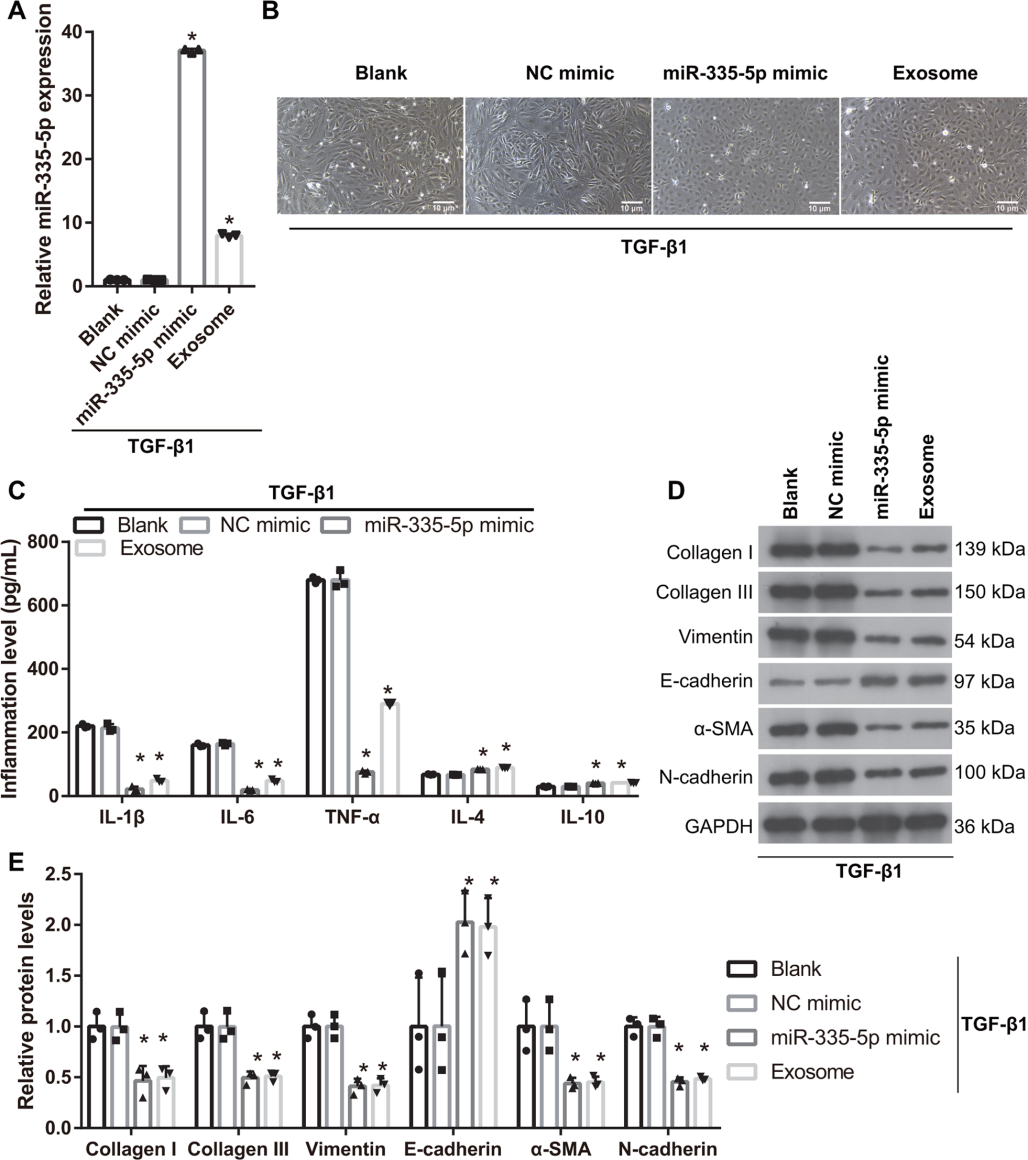
miR-335-5p overexpression can reverse the effect of TGF-β1 on the epithelial–myofibroblast transdifferentiation (EMT) and inflammation of HK-2 cells. **A** miR-335-5p expression was measured by RT-qPCR in TGF-β1-treated HK-2 cells after miR-335-5p mimic transfection (*n* = 3). **B** The shape of TGF-β1-treated HK-2 cells returned to a normal HK-2 cell morphology after transfection in TGF-β1-treated HK-2 cells (*n* = 3). **C** The protein levels of TNF-α, IL-6, and IL-1β were decreased whereas the levels of IL-4 and IL-10 were increased after transfection in TGF-β1-treated HK-2 cells. **D** The protein levels of collagen I, collagen III, α-SMA, vimentin, and N-cadherin in TGF-β1-induced HK-2 cells were decreased, whereas those of E-cadherin were increased, after miR-335-5p mimic transfection in TGF-β1-treated HK-2 cells (*n* = 3). **E** The statistical results of protein expression (*n* = 3). (*P < 0.05, vs NC mimic group). In exosome group, HK-2 cells were treated with TGF-β1 for 24 h and then TGF-β1-induced HK-2 cells were treated one-time 40 µg protein-equivalent of transfected-hucMSC-derived exosomes for 24 h. All statistical analyses were performed by one-way ANOVA, followed by Tukey’s multiple comparisons post hoc test

To further understand the function of miR-335-5p in the EMT of HK-2 cells induced by TGF-β1, the miR-335-5p mimic was transfected into HK-2 cells. The expression of miR-335-5p in the miR-335-5p mimic group was significantly higher than that in the NC mimic group (Fig. 5A). Compared with that in the NC mimic group, the shape of TGF-β1-induced HK-2 cells in the miR-335-5p mimic group changed from long spindles to cobblestone, similar to the result in the hucMSC-exosome treatment group (Fig. 5B); the protein levels of TNF-α, IL-6, IL-1β, collagen I, collagen III, α-SMA, vimentin, and N-cadherin were significantly decreased, whereas those of IL-4, IL-10, and E-cadherin were significantly increased in the miR-335-5p mimic group, similar to the result of the hucMSC-exosome treatment group (Fig. 5C–E).

### *ADAM19* is targeted by miR-335-5p

**Fig. 6:**
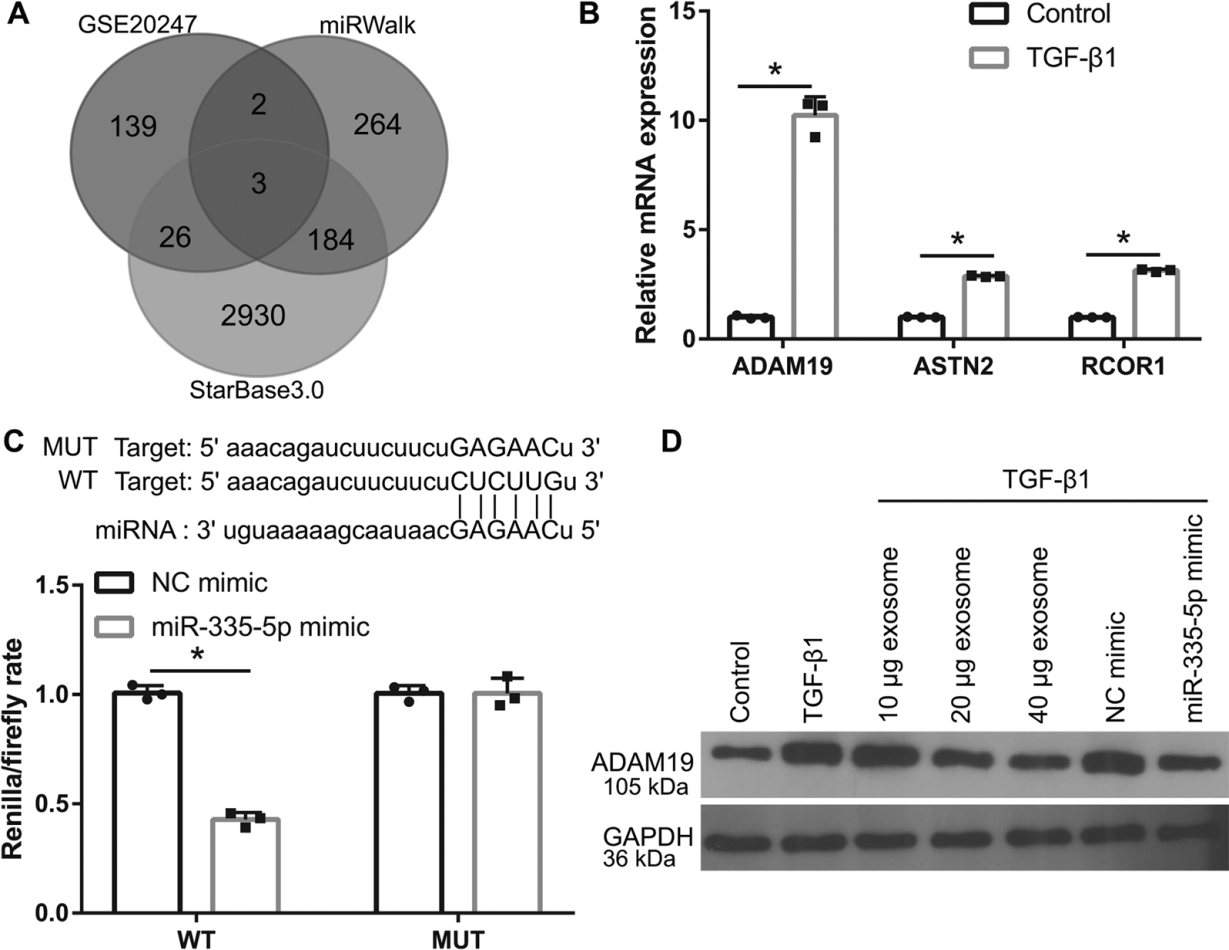
ADAM19 expression is inhibited by human umbilical cord mesenchymal stem cell (hucMSC)-exosome treatment in TGF-β1-treated HK-2 cells. **A** Venn image showing the overlapping targeted genes among StarBase3.0, miRWalk, and GSE20247 dataset analyses. **B** The expression of *ADAM19*, *ASTN2*, and *RCOR1* was measured by RT-qPCR in TGF-β1-treated HK-2 cells (*n* = 3). **C** The binding site of the *ADAM19* 3′-UTR and miR-335-5p is shown, and their binding was verified by luciferase analysis. **D** The protein level of ADAM19 in TGF-β1-treated HK-2 cells, measured by western blotting, was inhibited by hucMSC-exosome treatment or miR-335-5p mimic transfection (*n* = 3). (*P < 0.05, vs NC mimic group). All statistical analyses were performed by student's *t* test

StarBase3.0 and miRWalk analyses showed that there were 3143 and 453 potential target genes of miR-335-5p, respectively. Furthermore, the GSE20247 dataset showed that the expressions of 170 mRNAs were significantly upregulated in TGF-β1-treated HK-2 cells compared to those in normal HK-2 cells. The intersection of the three sets of results revealed three potential target genes, *ADAM19*, *ASTN2*, and *RCOR1* (Fig. 6A). Compared with that in normal cultured HK-2 cells, the expression of *ADAM19*, *ASTN2*, and *RCOR1* in TGF-β1-treated HK-2 cells was increased significantly, among which *ADAM19* expression increased the most (Fig. 6B). Hence, ADAM19 was selected for follow-up research. The binding site between the ADAM19 3′-UTR and miR-335-5p is shown in Fig. 5C. Luciferase analysis showed that compared with that in the NC + WT-ADAM19 3′-UTR group, the fluorescence activity in the miR-335-5p + WT-ADAM19 3′-UTR group was significantly reduced, whereas there was no significant difference between the NC + Mut-ADAM19 3′-UTR and miR-335-5p + Mut-ADAM19 3′-UTR groups, indicating that miR-335-5p can bind to the WT-ADAM19 3′-UTR (Fig. 6C). In addition, compared with those in normal HK-2 cells, ADAM19 protein levels in TGF-β1-treated HK-2 cells were significantly increased, which could be reversed by hucMSC-exosome treatment. Notably, compared with those in the NC mimic group, ADAM19 protein levels in TGF-β1-treated HK-2 cells were inhibited in the miR-335-5p mimic group (Fig. 6D).

### Downregulation of ADAM19 ameliorates the EMT and inflammation in HK-2 cells induced by TGF-β1 treatment

**Fig. 7:**
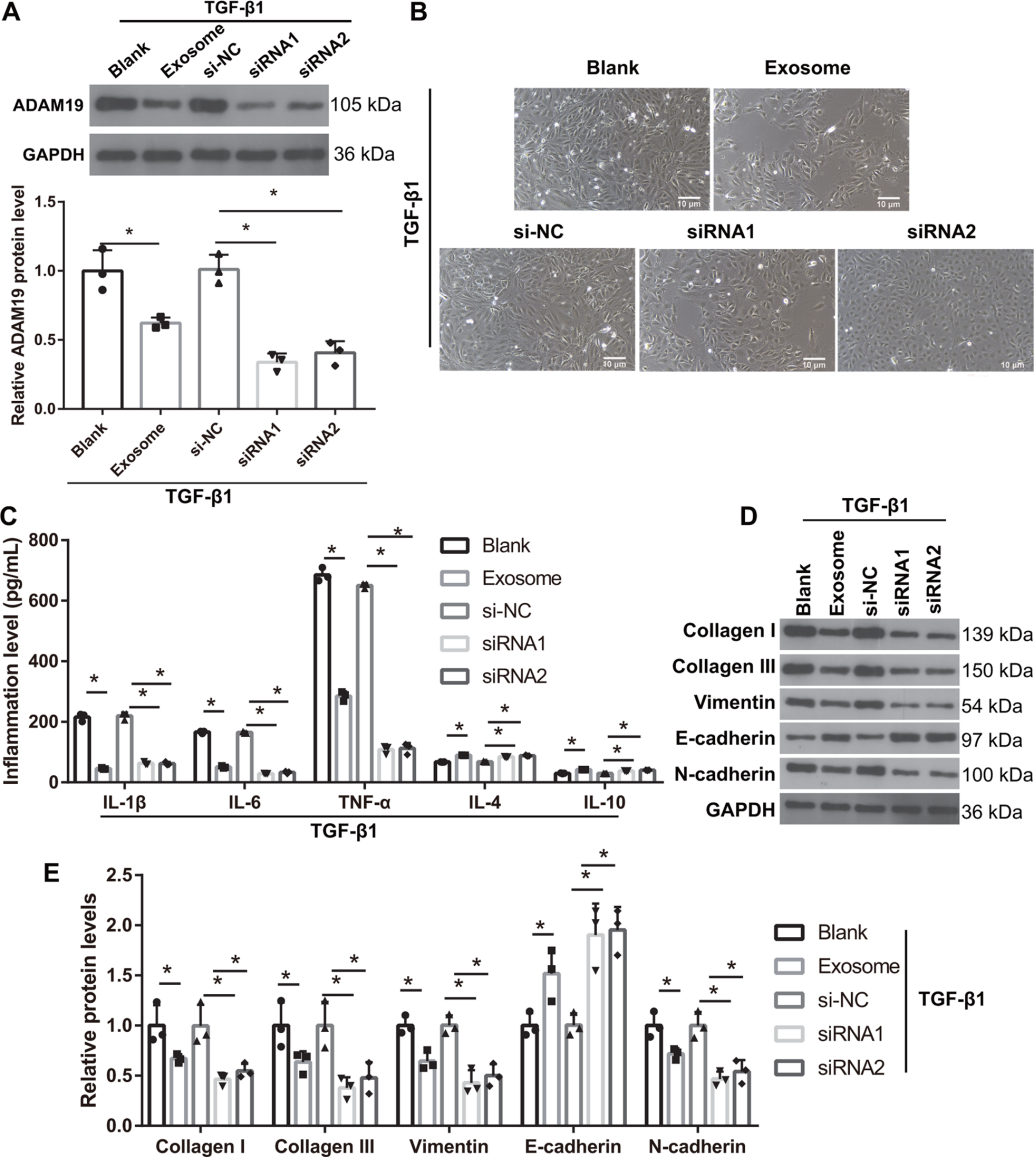
The absence of ADAM19 expression can reverse the effect of TGF-β1 on the epithelial–myofibroblast transdifferentiation (EMT) and inflammation of HK-2 cells. **A** ADAM19 protein levels were measured by western blotting in TGF-β1-treated HK-2 cells after si-ADAM19 transfection (*n* = 3). **B** The morphology of TGF-β1-treated HK-2 cells returned to normal after si-ADAM19 transfection (*n* = 3). **C** The protein levels of TNF-α, IL-6, and IL-1β were decreased whereas the levels of IL-4 and IL-10 were increased after si-ADAM19 transfection in TGF-β1-treated HK-2 cells (*n* = 3). **D** The protein levels of collagen I, collagen III, α-SMA, vimentin, and N-cadherin in TGF-β1-induced HK-2 cells were decreased, whereas those of E-cadherin were increased, after si-ADAM19 transfection in TGF-β1-treated HK-2 cells (*n* = 3). **E** The statistical results of protein expression (*n* = 3). *P < 0.05. In exosome group, HK-2 cells were treated with TGF-β1 for 24 h and then TGF-β1-induced HK-2 cells were treated one-time 40 µg protein-equivalent of transfected-hucMSC-derived exosomes for 24 h. All statistical analyses were performed by one-way ANOVA, followed by Tukey’s multiple comparisons post hoc test

To understand the effect of ADAM19 on the EMT of HK-2 cells induced by TGF-β1, si-ADAM19 was transfected into TGF-β1-induced HK-2 cells. Compared with those in the si-NC group, ADAM19 protein levels were significantly reduced in the si-ADAM19-1 and si-ADAM19-2 groups (Fig. 7A). Further research results showed that the morphology of TGF-β1-induced HK-2 cells in the si-ADAM19-1 and si-ADAM19-2 groups changed from long spindles to cobblestone (Fig. 7B). Compared with those in the si-NC group, the protein levels of TNF-α, IL-6, IL-1β, collagen I, collagen III, α-SMA, and N-cadherin were significantly reduced, whereas IL-4, IL-10, and E-cadherin protein was significantly increased in the si-ADAM19-1 and si-ADAM19-2 groups, similar to that observed in the hucMSC-exosome treatment group (Fig. 7C–E).

### ADAM19 overexpression reverses the effect of miR-335-5p on EMT in TGF-β1-induced HK-2 cells

**Fig. 8:**
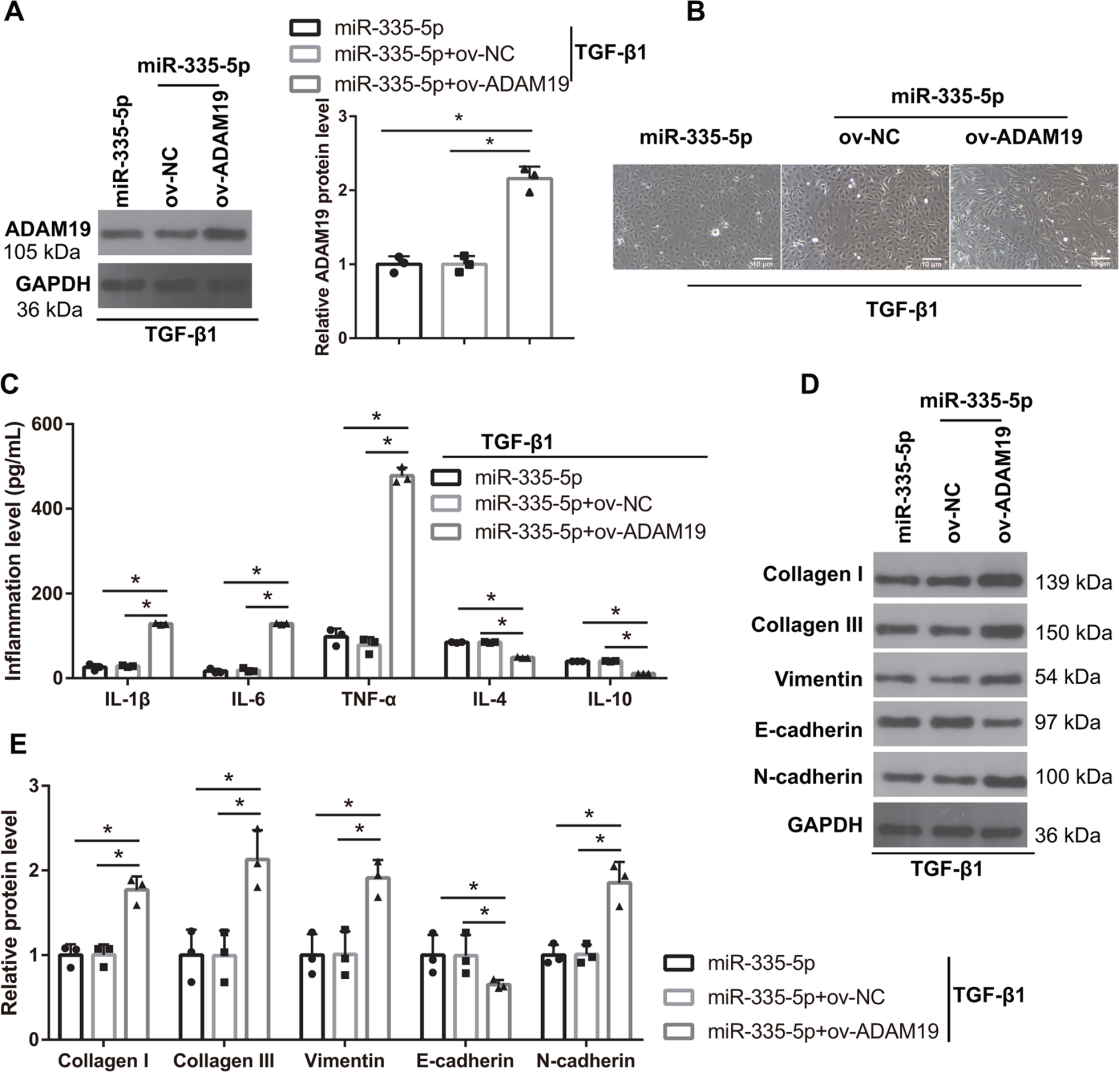
ADAM19 overexpression reverses the effect of miR-335-5p on epithelial–myofibroblast transdifferentiation (EMT) and inflammation in TGF-β1-induced HK-2 cells. **A** ADAM19 protein levels were measured by western blotting in TGF-β1-treated HK-2 cells after co-transfection of the miR-335-5p mimic and ov-ADAM19 (*n* = 3). **B** The shape of TGF-β1-treated HK-2 cells changed from a normal HK-2 cell morphology to a long spindle shape after co-transfection of the miR-335-5p mimic and ov-ADAM19 in TGF-β1-treated HK-2 cells (*n* = 3). **C** The levels of TNF-α, IL-6, and IL-1β were increased whereas the levels of IL-4 and IL-10 were decreased after co-transfection of the miR-335-5p mimic and ov-ADAM19 in TGF-β1-treated HK-2 cells (*n* = 3). **D** The protein levels of collagen I, collagen III, α-SMA, vimentin, and N-cadherin in TGF-β1-induced HK-2 cells were increased, whereas E-cadherin protein level was decreased after co-transfection of the miR-335-5p mimic and ov-ADAM19 in TGF-β1-treated HK-2 cells (*n* = 3). **E** The statistical results of protein expression (*n* = 3). All statistical analyses were performed by one-way ANOVA, followed by Tukey’s multiple comparisons post hoc test. *P < 0.05

To further understand whether miR-335-5p can regulate the EMT of TGF-β1-induced HK-2 cells through ADAM19, a miR-335-5p mimic and ov-ADAM19 were co-transfected into TGF-β1-induced HK-2 cells. Compared with those in the miR-335-5p mimic + ov-NC group, ADAM19 protein levels were significantly higher (Fig. 8A). The morphology TGF-β1-induced HK-2 cells changed from cobblestone to long spindles (Fig. 8B), and the protein levels of TNF-α, IL-6, IL-1β, collagen I, collagen III, α-SMA, and N-cadherin were significantly increased, whereas IL-4, IL-10, and E-cadherin protein was significantly reduced in the miR-335-5p mimic + ov-ADAM19 group (Fig. 8C–E).

## Discussion

Excessive extracellular matrix deposition and chronic inflammation can lead to renal fibrosis, end-stage renal disease, and renal failure [26]. The TGF-β1/Smad signaling pathway causes inflammation, EMT, and abnormal extracellular matrix deposition to promote renal fibrosis [7]. In this study, the shape of HK-2 cells changed from a cobblestone morphology to a long spindle shape after TGF-β1 treatment. Additionally, TGF-β1 treatment promoted cellular matrix deposition (as evidenced by enhanced collagen I and collagen III protein levels), inflammatory cytokines (TNF-α, IL-6, IL-1β), and EMT (as evidenced by increased the protein levels of α-SMA, vimentin, and N-cadherin and decreased protein levels of E-cadherin) and regulated anti-inflammatory cytokines (IL-4 and IL-10). In agreement with previous reports, these results suggested that TGF-β1 treatment can promote the EMT and inflammation of human RTECs.

Studies have shown that hucMSC-exosomes contain many different miRNAs compared to other cell-derived exosomes [25, 27]. HucMSC-exosomes can improve many diseases by transferring miRNAs. HucMSC-exosomal miR-377-3p can reduce lung epithelial cell inflammation and improve LPS-induced acute lung injury [25]. hucMSC-exosomes inhibit the inflammation of human colorectal mucosa cells by transferring miR-326 and improve inflammatory bowel disease [27]. hucMSC-exosomal miRNA-126-3p can enhance the vascularization of HUVECs and improve the surgical effect after vein transplantation [28]. This study found that the level of miR-335-5p in hucMSC-exosomes was high and that it could be transferred into TGF-β1-induced HK-2 cells. miR-335-5p is expressed at low levels in patients with liver fibrosis; thus, restoring the expression of miR-335-5p can inhibit the migration and activation of hepatic stellate cells and alleviate liver fibrosis [29]. Silencing the TGF-β1/Smad pathway can restore miR-335-5p expression and improve the activation and fibrosis of hepatic stellate cells [30]. miR-335-5p can inhibit the expression of SOS1, Smad2/3, and CTNNB1 proteins to exert anti-fibrotic effects in human gingival fibroblasts [31]. These studies indicate that miR-335-5p exerts an anti-fibrotic effect and can be inhibited by the TGF-β1/Smad signaling pathway. Similar to the result of a previous project, the results of this study indicate that the expression of miR-335-5p is reduced in HK-2 cells after TGF-β1 treatment. This study found that miR-335-5p can be transferred into TGF-β1-induced HK-2 cells via hucMSC-exosomes to attenuate EMT and inflammation and enhance anti-inflammatory cytokines expression. The overexpression of miR-335-5p in HK-2 cells also attenuated the EMT and inflammation and enhanced anti-inflammatory cytokines expression in TGF-β1-induced HK-2 cells. These results indicate that hucMSC-exosomes alleviate the TGF-β1-induced inflammation and EMT of HK-2 by transferring miR-335-5p. Although miR-335-5p exerts anti-EMT and inflammatory effects, miR-335-5p is easily degraded when it is externally injected into the body. And the viral vector used to carry miR-335-5p is easy to cause immune response. Whereas, exosome-transferred miR-335-5p may have greater advantages than miR-335-5p alone. Because exosome has various adhesion proteins on the surface and is the naturally occurring secretory vesicles with low toxicity, which have good tolerance and homing ability and are easily absorbed by the membrane in vivo [32–34].

ADAM19 plays a role in cell–cell and cell–matrix interactions [35]. ADAM19 expression is increased in TGF-β1-induced alveolar epithelial cells [36] and is significantly associated with the TGF-β1 signaling pathway and the activation of cardiac fibroblasts and cardiac fibrosis [37]. A previous study found that ADAM19 was primarily expressed in renal proximal tubular epithelial cells [38]. High ADAM19 expression is associated with glomerular fibrosis and inflammation [39]. Moreover, ADAM19 expression is increased in TGF-β1-induced renal cells and promotes renal EMT [35]. In this study, ADAM19 expression was also increased in TGF-β1-induced HK-2 cells. Additionally, ADAM19 was found to be the target gene of miR-335-5p, and miR-335-5p treatment could reduce the protein levels of ADAM19. The inhibition of ADAM19 expression was determined to attenuate EMT and inflammation and enhance anti-inflammatory cytokines expression in TGF-β1-induced HK-2 cells, and ADAM19 overexpression counteracted the effect of miR-335-5p on EMT and inflammation in TGF-β1-induced HK-2 cells. These results indicated that miR-335-5p improves the EMT and inflammation phenotypes of TGF-β1-induced HK-2 cells by reducing ADAM19 protein levels.

However, there are three limitations to the present study. Apart from miR-335-5p, other miRNAs might exert a regulatory effect on HK-2 cells. The expression and effect of miR-335-5p and ADAM19 during renal fibrosis in vivo require further study. Furthermore, the downstream signaling pathways regulated by ADAM19 need further verification.

## Conclusion

miR-335-5p and ADAM19 are involved in the TGF-β-induced EMT and inflammation in HK-2 cells. HucMSC-derived exosomal miR-335-5p exerts anti-EMT and anti-inflammatory effects in TGF-β1-induced HK-2 cells by reducing ADAM19 protein levels. This study provides a potential clinical therapeutic strategy and targets for the treatment of renal fibrosis.

## Data Availability

All data used during the study are shown in this manuscript.
